# The Nation's Food and the War

**Published:** 1917-03-03

**Authors:** 


### The Hospital

The Workers' Newspaper of Administrative Medicine and Institutional Life, Administration, National Insurance and Health.

No. 1604, Vol. LXI. SATURDAY, MARCH 3, 1917. PRICE ONE PENNY.

### THE NATION'S FOOD AND THE WAR.

In any large and comprehensive sense the food question has never in recent years much concerned the British public. The neglect has not been due to any failure in the numbers of the prophets. On the contrary, these have been numerous, and they have spoken really terrible things. But partly in consequence of a conflict between their gloomy threatenings and the apparent teachings of experience, and partly because of their mutual contradictions, the prophets have never really gained the attention or confidence of the crowd. Broadly, we are inclined to say that the crowd has been right and the prophets wrong, for, in the last resort, it is less by laboratory experiments and chemical calculations than by the accumulated experiences of the race that a diet suitable for vigorous and enterprising national life should be determined. The physiologists may help us with explanations and suggestions, but it is not with them that there rests the last word. Food and feeding have influences other than those which determine mere animal nutrition, and the contribution they make to these ends cannot be weighed in a balance or expressed in a formula. Indeed, the relation between the one and the other is rather felt as an instinct than appreciated as an argument. It is this instinct which has proved the successful opponent of the scientific and pseudo-scientific expert.

A kindred experience can be related of food in reference to national security, though here more tangible motives are responsible for the failure of the seers. Again and again the nation has been warned that grave danger was involved in an arrangement by which, of the total food consumption of the country, only one-third was produced at home. To accumulate stores for adverse times was one prescription, to develop the science and craft of agriculture was another. But with abundant food supplies brought from the ends of the earth, and with cheapness in the markets, neither one form of advice nor the other received much practical support. War was regarded as improbable, or, in the event of its occurrence, the seas would be kept open and free by the British Navy even as in times of peace. From these smooth dreams the events of the last few weeks and the recent speech of the Prime Minister have produced a rude awakening. The nation is compelled to recognise that food, so far from being a matter for careless unconcern, is of the first importance in the struggle towards victory. Neither the people nor the experts foresaw the full meaning of the submarine weapon, and still less did they anticipate the existence of the submarine soul. But both the one and the other are at our doors, and, as a consequence, food in its most primitive values demands urgent attention if national disaster is to be avoided.

The position admittedly is an anxious one, but it will not be helped by exaggeration, and still less is it helped by the people who tell us that we are to suffer because we neglected their advice. It may be so, but over vain regrets we have no time to waste. Clearly there are only two alternatives. Either supplies must be increased or consumption must be diminished. It is on purely practical grounds—with politics we have here no concern—that we find ourselves in cordial sympathy with the emphasis laid by the Prime Minister on the former of these two methods. Not that we offer any objection to the plain hints of the Food Controller or question for one moment the duty of patriotic obedience to his behests. But we cannot believe that any considerable diminution of the common articles of diet can be achieved without prejudice to the productive energy of the nation. That individuals in certain classes of society—perhaps in all classes—eat too much is doubtless true, but we are convinced that no such proposition is true of the great mass of the industrial workers upon whose thews and sinews the cause of the country now directly depends. That they may be kept alive with less food than they now consume is beyond challenge, but will they, in such circumstances, display the vigour and good will that are holding high the spirit of the nation and the flexibility and force which arm our forces for the fray? It is not merely that life may be preserved to the citizens that the nation has need of an abundant food supply. Safety demands something more—namely, enterprise to undertake, energy to accomplish, and courage to endure; and between these attainments and abundant nutrition there is for the mass of people a closer relation than the sentimentalist is ready to recognise. That our people, if put to it, will, in spite of insufficient feeding, carry on we do not doubt. But equally we are sure that the present high level of energy would in such circumstances begin to fail and flag, and though endurance might be maintained, accomplishment would touch a lower note. Here, it seems to us, are the essential motives why, so far as such a method is possible, the food problem of the immediate future should look for solution to increased supplies rather than to diminished consumption.

There is, however, plenty of opportunity for auxiliaries. Thus the agencies which are setting out practical schemes translating into daily meals the injunctions of the Food Controller deserve every sympathy and encouragement; in the present hour they are much more helpful than those whose talk is ever of calories and of proximate principles. Last, but by no means least, is the economy and preparation of food in our households, and here, obviously, the opportunity is with the women. The work will not secure the blaze of the limelight, but it is of high patriotic value, and to true hearts this is the best appeal. By all means in these and kindred ways let saving be encouraged and practised. But without neglecting this aspect of the question, it is to an increase of supplies that the effort of our leaders should be mainly directed. Physiology in the widest sense of this term supports what is urged in the sphere of affairs on political and economic grounds. Unless the vigour of the nation is to decline a full supply of the essential foods must certainly be secured.

